# Hepatocellular Carcinoma in Metabolic Dysfunction-Associated Steatotic Liver Disease

**DOI:** 10.1001/jamanetworkopen.2024.21019

**Published:** 2024-07-11

**Authors:** Luis A. Rodriguez, Julie A. Schmittdiel, Liyan Liu, Brock A. Macdonald, Sreepriya Balasubramanian, Krisna P. Chai, Suk I. Seo, Nizar Mukhtar, Theodore R. Levin, Varun Saxena

**Affiliations:** 1 Division of Research, Kaiser Permanente Northern California, Oakland; 2 Department of Health Systems Science, Kaiser Permanente Bernard J. Tyson School of Medicine, Pasadena, California; 3 Department of Epidemiology & Biostatistics, University of California, San Francisco; 4 Kaiser Permanente San Rafael Medical Center, San Rafael, California; 5 Kaiser Permanente Roseville Medical Center, Roseville, California; 6 Kaiser Permanente Santa Clara Homestead Medical Center, Santa Clara, California; 7 Kaiser Permanente Walnut Creek Medical Center, Walnut Creek, California; 8 Kaiser Permanente San Francisco Medical Center, San Francisco, California; 9 Kaiser Permanente South San Francisco Medical Center, South San Francisco, California; 10 Department of Gastroenterology and Transplant Hepatology, University of California, San Francisco

## Abstract

**Importance:**

In the US, hepatocellular carcinoma (HCC) has been the most rapidly increasing cancer since 1980, and metabolic dysfunction–associated steatotic liver disease (MASLD) is expected to soon become the leading cause of HCC.

**Objective:**

To develop a prediction model for HCC incidence in a cohort of patients with MASLD.

**Design, Setting, and Participants:**

This prognostic study was conducted among patients aged at least 18 years with MASLD, identified using diagnosis of MASLD using *International Classification of Diseases, Ninth Revision (ICD-9)* or *International Statistical Classification of Diseases and Related Health Problems, Tenth Revision (ICD-10)* diagnosis codes; natural language processing of radiology imaging report text, which identified patients who had imaging evidence of MASLD but had not been formally diagnosed; or the Dallas Steatosis Index, a risk equation that identifies individuals likely to have MASLD with good precision. Patients were enrolled from Kaiser Permanente Northern California, an integrated health delivery system with more than 4.6 million members, with study entry between January 2009 and December 2018, and follow-up until HCC development, death, or study termination on September 30, 2021. Statistical analysis was performed during February 2023 and January 2024.

**Exposure:**

Data were extracted from the electronic health record and included 18 routinely measured factors associated with MASLD.

**Main Outcome and Measures:**

The cohort was split (70:30) into derivation and internal validation sets; extreme gradient boosting was used to model HCC incidence. HCC risk was divided into 3 categories, with the cumulative estimated probability of HCC 0.05% or less classified as low risk; 0.05% to 0.09%, medium risk; and 0.1% or greater, high risk.

**Results:**

A total of 1 811 461 patients (median age [IQR] at baseline, 52 [41-63] years; 982 300 [54.2%] female) participated in the study. During a median (range) follow-up of 9.3 (5.8-12.4) years, 946 patients developed HCC, for an incidence rate of 0.065 per 1000 person-years. The model achieved an area under the curve of 0.899 (95% CI, 0.882-0.916) in the validation set. At the medium-risk threshold, the model had a sensitivity of 87.5%, specificity of 81.4%, and a number needed to screen of 406. At the high-risk threshold, the model had a sensitivity of 78.4%, a specificity of 90.1%, and a number needed to screen of 241.

**Conclusions and Relevance:**

This prognostic study of more than 1.8 million patients with MASLD used electronic health record data to develop a prediction model to discriminate between individuals with and without incident HCC with good precision. This model could serve as a starting point to identify patients with MASLD who may need intervention and/or HCC surveillance.

## Key Points

**Question:**

In patients with metabolic dysfunction–associated steatotic liver disease (MASLD), can those at high risk of developing hepatocellular carcinoma (HCC) be identified?

**Findings:**

This prognostic study used electronic health record data from 1 811 461 patients with MASLD in an integrated health care delivery system in Northern California to develop a prediction model to discriminate between individuals with and without incident HCC with good precision.

**Meaning:**

These findings suggest that this model could serve as a starting point to identify patients with MASLD at high risk of HCC and guide decision-making about risk stratifying patients for prevention efforts and/or HCC surveillance.

## Teaser

This prognostic study describes the development and performance of a risk stratification model to identify patients with metabolic dysfunction–associated steatotic liver disease at high risk of developing hepatocellular carcinoma.

## Introduction

In the US, hepatocellular carcinoma (HCC) has been the most rapidly increasing cancer since 1980.^1^ The rate of death from HCC increased by 43% (from 7.2 to 10.3 deaths per 100 000 population) between 2000 and 2016.^1^ With a 5-year survival of 18%, HCC is the second most lethal malignant neoplasm after pancreatic cancer.^1^ In North America, among patients with HCC, the primary risk factors include alcohol (37% of patients), hepatitis C virus (HCV; 31% of patients), hepatitis B (HBV; 9% of patients), and other causes, including metabolic dysfunction–associated steatotic liver disease (MASLD) (23% of patients).^2^ However, with the implementation of universal HBV vaccination in newborns in many countries,^3,4^ HCV treatment programs worldwide, and increasing prevalence of overweight and obesity,^5^ the epidemiology of HCC is shifting away from viral hepatitis to metabolic dysfunction–associated steatohepatitis.^2^ Due to uncertainty about the benefits of universal HCC screening or surveillance, it is not recommended in patients with noncirrhotic MASLD.^2^ However, currently, up to 30% of patients with MASLD-related HCC do not have cirrhosis.^6^ With the increasing prevalence,^7^ it is projected that MASLD will soon become the leading cause of HCC in the US,^2^ which has implications for how physicians and health care systems should prioritize surveillance of patients with MASLD. Nevertheless, universally screening all patients with MASLD would be challenging, due to a lack of adequate imaging resources.^8^ A risk stratification model may allow those at highest risk for HCC to receive the most effective screening.

Prior studies have identified unique MASLD-related HCC risk factors, including diabetes, obesity, metabolic syndrome, alcohol, smoking, increased gut permeability, altered microbiome composition, and Hispanic ethnicity.^8,9^ In addition, genome-wide studies have identified variations in genetic makeup that also contribute to MASLD risk.^8^ Despite known MASLD-related HCC risk factors, few prediction models have been developed in MASLD populations,^7,10,11,12^ primarily using genetic risk scores,^10^ limiting their applicability in routine clinical settings, or in racially and ethnically homogeneous populations,^11,12,13^ limiting their generalizability in racially and ethnically diverse populations.

Thus, there is a need to develop risk stratification tools using routinely collected demographic and clinical variables from diverse populations in clinical settings to identify a subgroup of patients with high-risk MASLD with and without cirrhosis in whom HCC surveillance can be prioritized.^8^ To address this gap, we developed a prediction model for HCC incidence using routinely measured data from the electronic health record (EHR) from a large multiracial and multiethnic cohort of patients with MASLD.

## Methods

This prognostic study was approved by the Kaiser Permanente Northern California (KPNC) institutional review board, which waived the requirement for informed consent for study individuals, given the use of only electronic health record (EHR) data. We followed the Transparent Reporting of a Multivariable Prediction Model for Individual Prognosis or Diagnosis (TRIPOD) reporting guideline for model derivation and internal validation.

### Study Population and Data Source

This is a prognostic study of 1 811 461 adult patients (age ≥18 years) with MASLD. Study entry was between January 1, 2009, and December 31, 2018, and we retrospectively followed up patients until HCC development, disenrollment, death, or study termination on September 30, 2021. The study population was derived from KPNC, an integrated health delivery system with more than 4.6 million members.^14^ To ensure complete data for model development, we excluded anyone with a noncontinuous KPNC membership during follow-up, where noncontinuous was defined as the first nonmember gap of at least 3 months within 24 months after study entry.^15^ Entry into the study was on the date of prevalent MASLD ascertainment using any of the following 3 methods^16^ (eTable 1 in Supplement 1): diagnosis of MASLD using *International Classification of Diseases, Ninth Revision (ICD-9)* or *International Statistical Classification of Diseases and Related Health Problems, Tenth Revision (ICD-10)* diagnosis codes; natural language processing of radiology imaging report text, which identified some patients who had imaging evidence of MASLD but had not been formally diagnosed; and likely to have MASLD but without a diagnosis, using the Dallas Steatosis Index (DSI).^17^ The DSI is a risk equation based on the following demographic and clinical variables to identify MASLD: sex, age, race and ethnicity, body mass index (BMI; calculated as weight in kilograms divided by height in meters squared), fasting triglycerides, alanine aminotransferase (ALT), hypertension, diabetes, and fasting glucose. To calculate the DSI, we first identified a fasting triglyceride level and then identified ALT, BMI, and fasting blood glucose levels within 1 year before through 6 months after the date of triglyceride record. If multiple measurements were found, we kept the one closest to the triglyceride measurement. The remaining variables were ascertained on the same triglyceride date (study entry date). We found that the application of these 3 methods across a large population yielded MASLD prevalence close to expected based on data from epidemiologic studies.^18,19^

Patients who had an HCC diagnosis before study entry were excluded. To improve the identification of MASLD, patients with the following diagnoses were excluded: HCV infection, autoimmune hepatitis, primary biliary cirrhosis, acute alcoholic hepatitis, alcoholic fatty liver, unspecified alcoholic liver damage, alcoholic cirrhosis, alcohol use disorder, and chemical dependency treatment (*ICD-9* and *ICD-10* diagnostic codes are provided in eTable 1 in Supplement 1).

### Baseline Patient Characteristics

Baseline characteristics were ascertained at the time of cohort entry. All data were extracted from the EHR. We selected 18 predictors a priori from published literature linking them to HCC risk.^2,8,9,11,20,21,22,23,24,25,26^ The following variables were included: patient demographics (age, sex, race and ethnicity), anthropometric measure (BMI), substance use (smoking status, drug use), comorbid conditions (diabetes, HIV, HBV, cirrhosis, steatohepatitis, other autoimmune disease), and laboratory data (platelets, albumin, ALT, aspartate transaminase [AST], total bilirubin, and international normalized ratio [INR]).

### Predictor and Outcome Measurements

All predictors were collected at baseline. Age was included as a continuous measure. Sex was categorized into female and male, and race and ethnicity were collected from various member surveys and registries using EHR data (which include self-reported, insurance files, and clinician-observed). Those with Hispanic ethnicity were identified first and the remaining participants were categorized into Asian (including Native Hawaiian and Pacific Islander), Black, White, and other (including American Indian or Alaskan Native, missing or unknown, or multiracial). BMI was included as a continuous variable. Smoking history was categorized as current, former, never, and unknown. Diabetes was identified using the KPNC Diabetes Registry, which uses diagnoses, laboratory tests, and prescriptions for antihyperglycemic medications.^27^ Drug use was identified using ICD-9 and *International Classification of Diseases, Ninth Revision, Clinical Modification (ICD-9-CM)* diagnosis codes (eTable 2 in Supplement 1). Comorbid conditions (HIV, steatohepatitis, other autoimmune disease) were identified using *ICD-9* and *ICD-10-CM* diagnosis codes, HBV using antigen-positive status and/or detectable or quantifiable HBV DNA levels, and cirrhosis was identified using *ICD-9* and *ICD-10-CM* diagnosis codes,^28^ or using a Fibrosis-4 calculator (FIB-4) score of 3.25 or greater.^29^ The diagnosis of HCC was captured using the KPNC Division of Research Cancer Registry^30^ and retrospectively using death certificates.

### Missing Data and Indicators

Those with missing data for race and ethnicity and smoking status were included with the other race and ethnicity category and the unknown smoking history category, respectively. Missing data for BMI (6% of sample) was imputed at the predictive mean using R package multivariate imputation using chained equations.^31^ Clinical variables had varying degrees of missingness ranging from 4% for ALT, 17% for platelets, 45% for AST, 61% for bilirubin, 74% for INR, and 77% for albumin and were not missing at random; thus, we did not impute.^32^ Instead, these continuous variables were categorized into 5 groups, including quartile groups based on the 25th, 50th, and 75th percentiles, and a fifth missing category. All these variables, including their missing categories, were included in the development of the prediction algorithm.

### Statistical Analysis

Descriptive statistics were presented as median and IQR for continuous variables and proportions for categorical variables. The study population was randomly divided into 2 samples: derivation (70%) and validation (30%). An extreme gradient boosting algorithm was then used to model the risk of HCC using R package XGBoost.^33^ The follow-up period was from the index date to HCC development, loss of membership, or death or administrative censoring at study termination on September 30, 2021. The extreme gradient boosting algorithm is an ensemble of decision trees algorithms in which decision trees are created in sequential form. For each decision tree, a weight is added to all the independent variables to predict the outcome under study. In XGBoost, the gradient boosting algorithm is used to iteratively refine the model by adding new decision trees that correct the errors of the previous trees. Thus, the gradient boosting algorithm produces a robust predictive model. We used 10-fold cross-validation in the gradient boosting algorithm, and a total of 1141 rounds were run for the optimal results. The risk of developing HCC was divided into 3 categories, with the predicted probability of developing HCC of 0.05% or less classified as low risk; 0.05% to 0.09%, medium risk; and 0.1% or greater, high risk, over a median (range) follow-up of 9.3 (5.8-12.4) years (eFigure in Supplement 1). Stratified analyses were also performed by cirrhosis status, race and ethnicity groups, and age categories (≤40, 41-65, 66-75, ≥76 years). Statistical analysis was performed during February 2023 to January 2024. All analyses were completed in SAS version 9.4 (SAS Institute) and R version 3.4.1 (R Project for Statistical Computing).

**Figure 1.**
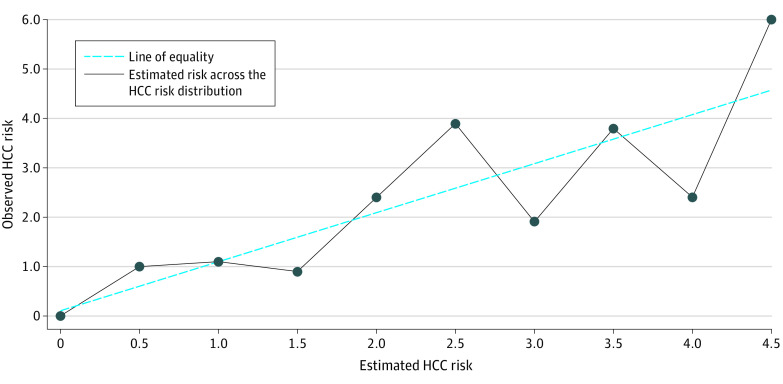
Calibration Plot Showing Mean Predicted vs Observed Hepatocellular Carcinoma (HCC) Risk in the Validation Set (n = 543 577)

Discrimination ability was evaluated using area under the receiver operating characteristic curve (AUC),^34^ sensitivity, specificity, positive predictive value (PPV), negative predictive value (NPV), and the number needed to screen (NNS) at the specified medium- and high-risk thresholds of HCC risk. Calibration performance was evaluated on the validation sample with the Hosmer-Lemeshow goodness-of-fit measure, by calculating the mean calibration (calibration in the large), the calibration slope, and graphically using a calibration plot at different points across the HCC risk distribution (Figure 1).^34,35^

## Results

### Characteristics of Study Population

Among 1 811 461 included patients (median age [IQR] at baseline, 52 [41-63] years; 982 300 [54.2%] female), patient characteristics were similar between the derivation and validation subsets (Table 1). and compared with patients without HCC they were older (median [IQR] age, 68 [61-75] vs 52 [41-63] years), had a higher BMI (median [IQR], 29.2 [25.9-33.7] vs 27.8 [24.4-32.1], were more likely to be male (63% vs 45.8%), were more likely to be Asian (27.8% vs 20.5%), and were less likely to be Black (3.2% vs 6.5%) or Hispanic (12.3% vs 15.2%). Patients with HCC were also more likely to have a diagnosis of cirrhosis (12.2% vs 0.9%), HBV (2.7% vs 1.2%), steatohepatitis (3.6% vs 1%), or other autoimmune disease (4.3% vs 0.1%). Patients with HCC had a lower distribution of platelets and albumin, and higher distributions of ALT, AST, and total bilirubin (Table 1).

**Table 1.** Study Participant Baseline Characteristics, Stratified by HCC Development in the Derivation and Validation Samples

Characteristic	Participants, No. (%)			
	Derivation sample		Validation sample	
	Non-HCC (n = 1 267 244)	HCC (n = 640)	Non-HCC (n = 543 271	HCC (n = 306)
Age, median (IQR), y	52 (41-63)	68 (61-75)	52 (41-63)	67 (60-74)
Sex				
Female	686 977 (54.2)	235 (36.7)	294 973 (54.3)	115 (37.6)
Male	580 267 (45.8)	405 (63.3)	248 298 (45.7)	191 (62.4)
Race and ethnicity				
Asian and Native Hawaiian and Pacific Islander	260 293 (20.5)	181 (28.3)	111 339 (20.5)	82 (26.8)
Black	82 882 (6.5)	17 (2.7)	35 775 (6.6)	13 (4.2)
Hispanic	192 452 (15.2)	78 (12.2)	82 564 (15.2)	38 (12.4)
White	647 304 (51.1)	309 (48.3)	277 894 (51.2)	142 (46.4)
Other	84 313 (6.7)	55 (8.6)	35 699 (6.6)	31 (10.1)
BMI, median (IQR)	27.8 (24.4-32.1)	28.9 (25.7-33.3)	27.8 (24.4-32.2)	29.9 (26.2-34.4)
Smoking history				
Current	88 346 (7)	32 (5)	37 774 (7)	18 (5.9)
Former	208 854 (16.5)	172 (26.9)	89 354 (16.4)	73 (23.9)
Never	695 944 (54.9)	215 (33.6)	298 774 (55)	111 (36.3)
Unknown	274 100 (21.6)	221 (34.5)	117 369 (21.6)	104 (34)
Diabetes	165 621 (13.1)	342 (53.4)	71 261 (13.1)	161 (52.6)
Drug use	5928 (0.5)	<10 (<1)	2538 (0.5)	<10 (<1)
HIV	1909 (0.2)	<10 (<1)	887 (0.2)	<10 (<1)
HBV	15 298 (1.2)	17 (2.7)	6759 (1.2)	9 (2.9)
Steatohepatitis	13 000 (1)	23 (3.6)	5471 (1)	<10 (<4)
Other autoimmune disease	851 (0.1)	23 (3.6)	363 (0.1)	18 (5.9)
Cirrhosis	11 406 (0.9)	79 (12.3)	4770 (0.9)	36 (11.8)
Platelets, ×10^3^/µL				
<210	255 947 (20.2)	302 (47.2)	109 804 (20.2)	129 (42.2)
210-247	265 977 (21.0)	93 (14.5)	114 727 (21.1)	53 (17.3)
248-290	260 406 (20.5)	55 (8.6)	111 181 (20.5)	34 (11.1)
>290	267 321 (21.1)	65 (10.2)	114 406 (21.1)	29 (9.5)
Missing	217 593 (17.2)	125 (19.5)	93 153 (17.1)	61 (19.9)
Albumin, g/dL				
<3.9	66 818 (5.3)	151 (23.6)	28 535 (5.3)	77 (25.2)
3.9-4.1	54 384 (4.3)	85 (13.3)	23 118 (4.3)	43 (14.1)
4.2-4.5	85 736 (6.8)	91 (14.2)	36 928 (6.8)	35 (11.4)
>4.5	80 618 (6.4)	69 (10.8)	34 506 (6.4)	34 (11.1)
Missing	979 688 (77.3)	244 (38.1)	420 184 (77.3)	117 (38.2)
ALT, U/L				
<14	269 050 (21.2)	69 (10.8)	115 293 (21.2)	20 (6.5)
14-18	306 520 (24.2)	94 (14.7)	131 204 (24.2)	40 (13.1)
19-28	335 424 (26.5)	140 (21.9)	143 973 (26.5)	74 (24.2)
>28	304 786 (24.1)	299 (46.7)	130 857 (24.1)	150 (49.0)
Missing	51 464 (4.1)	38 (5.9)	21 944 (4.0)	22 (7.2)
AST, U/L				
<17	148 860 (11.7)	27 (4.2)	64 141 (11.8)	13 (4.2)
17-20	176 261 (13.9)	41 (6.4)	75 402 (13.9)	26 (8.5)
21-28	199 296 (15.7)	103 (16.1)	85 552 (15.7)	41 (13.4)
>28	175 156 (13.8)	324 (50.6)	75 393 (13.9)	160 (52.3)
Missing	567 671 (44.8)	145 (22.7)	242 783 (44.7)	66 (21.6)
Total bilirubin, mg/dL				
<0.40	81 268 (6.4)	46 (7.2)	35 085 (6.5)	20 (6.5)
0.40-0.49	83 462 (6.6)	42 (6.6)	35 662 (6.6)	15 (4.9)
0.50-0.70	156 959 (12.4)	101 (15.8)	67 661 (12.5)	57 (18.6)
>0.70	175 123 (13.8)	259 (40.5)	74 887 (13.8)	117 (38.2)
Missing	770 432 (60.8)	192 (30.0)	329 976 (60.7)	97 (31.7)
INR				
<0.99	44 472 (3.5)	24 (3.8)	19 073 (3.5)	<10 (<4)
1.00-1.10	166 609 (13.1)	92 (14.4)	72 057 (13.3)	47 (15.4)
1.11-1.20	70 708 (5.6)	33 (5.2)	29 979 (5.5)	18 (5.9)
>1.20	52 491 (4.1)	19 (3.0)	22 655 (4.2)	15 (4.9)
Missing	932 964 (73.6)	472 (73.8)	399 507 (73.5)	216 (70.6)

Abbreviations: ALT, alanine transaminase; AST, aspartate transaminase; BMI, body mass index (calculated as weight in kilograms divided by height in meters squared); HBV, hepatitis B virus; HCC, hepatocellular carcinoma; INR, international normalized ratio.

SI conversion factors: To convert albumin to proportion of 1.0, multiply by 0.1; ALT and AST to microkatals per liter, multiply by 0.0167; bilirubin to micromoles per liter, multiply by 17.104; platelets to ×10^9^/L, multiply by 1.

### HCC Incidence

Among 1 811 461 patients, 946 (0.05%) developed HCC over a median (range) follow-up of 9.3 (5.8-12.4) years, for an incidence rate of 0.065 per 1000 person-years (Table 2). The 5-year cumulative incidence was 0.003% in the low-risk group, 0.018% in the medium-risk group, and 0.242% in the high-risk group. In stratified analyses, the HCC incidence rate was significantly greater in the subset with cirrhosis (1.216 events per 1000 person-years), compared with patients without cirrhosis (0.057 events per 1000 person-years). Asian patients had the highest HCC incidence (0.088 events per 1000 person-years); incidence was 0.03 events per 1000 person-years among Black patients, 0.054 events per 1000 person-years among Hispanic patients, 0.061 events per 1000 person-years among White patients, and 0.085 events per 1000 person-years among patients who identified as other race or ethnicity. The HCC incidence rate was higher in patients aged 76 years and older (0.204 events per 1000 person-years), followed by patients aged 66 to 75 years (0.181 events per 1000 person-years) and 41 to 65 years (0.045 events per 1000 person-years), and lowest among those aged 40 years or younger (0.005 events per 1000 person-years) (Table 2).

**Table 2.** Discrimination Between Patients With and Without HCC Using the Extreme Gradient Boosting Model, by Demographic and Clinical Characteristics

Characteristic	Sample size, No. (%)	Incident HCC cases	HCC incidence rate, per 1000 person-years	AUC (95% CI) of validation sample
Overall	1 811 461 (100)	946	0.065	0.899 (0.882-0.916)
Cirrhosis				
Yes	16 291 (0.9)	115	1.216	0.824 (0.740-0.908)
No	1 795 170 (99.1)	831	0.057	0.893 (0.875-0.911)
Race and ethnicity				
Asian and Native Hawaiian or Pacific Islander	371 895 (20.5)	263	0.088	0.924 (0.903-0.944)
Black	118 687 (6.6)	30	0.030	0.836 (0.734-0.938)
Hispanic	275 132 (15.2)	116	0.054	0.942 (0.909-0.975)
White	925 649 (51.1)	451	0.061	0.919 (0.901-0.937)
Other	120 098 (6.6)	86	0.085	0.887 (0.833-0.940)
Age category, y				
≤40	448 545 (24.8)	17	0.005	0.958 (0.931-0.986)
41-65	995 908 (55.0)	373	0.045	0.872 (0.837-0.908)
66-75	232 869 (12.9)	365	0.181	0.839 (0.798-0.879)
≥76	134 139 (7.4)	191	0.204	0.798 (0.740-0.855)

Abbreviations: AUC, area under the curve; HCC, hepatocellular carcinoma.

### HCC Predictors

**Figure 2.**
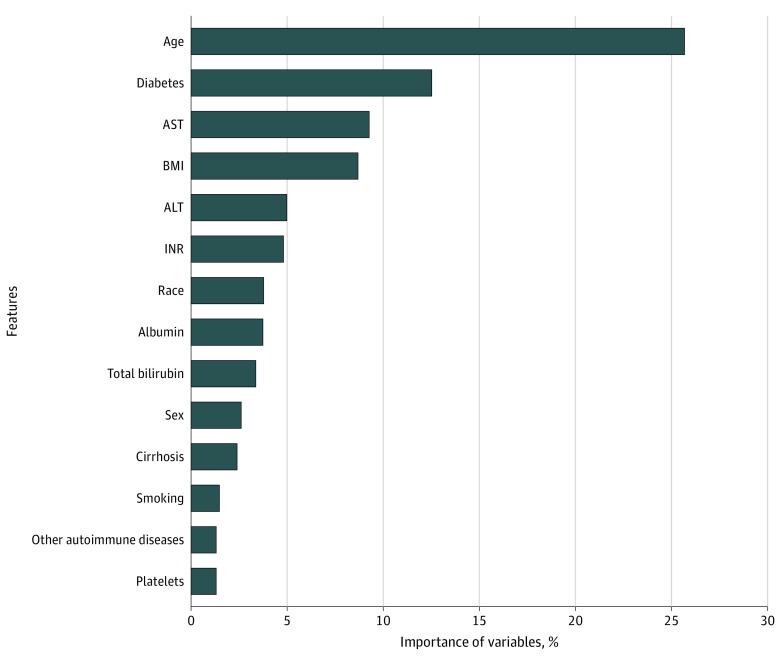
Relative Importance of Each Variable in the Extreme Gradient Boosted Model to Predict Hepatocellular Carcinoma Risk The variable importance measure is scaled to have a maximum value of 100%. ALT indicates alanine transaminase; AST, aspartate transaminase; BMI, body mass index; INR, international normalized ratio.

The extreme gradient boosting algorithm included 18 predictors. The top 10 predictors of HCC in terms of predictive importance or contribution included age, diabetes, AST, BMI, ALT, INR, race and ethnicity, albumin, total bilirubin, and sex (Figure 2). The bottom 8 predictors included cirrhosis, smoking, platelets, other autoimmune diseases, HBV, steatohepatitis, drug use, and HIV.

### Model Performance

**Figure 3.**
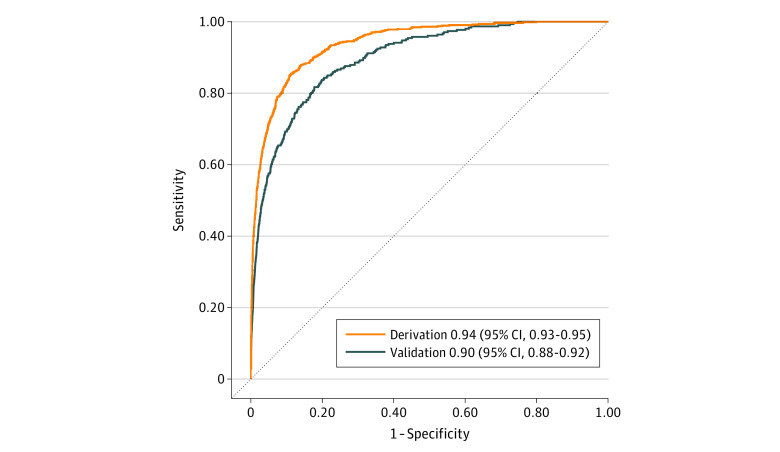
Area Under the Receiver Operating Characteristic Curve in the Derivation (n = 1 267 884) and Validation (n = 543 577) Samples

In the derivation set, the AUC was 0.941 (95% CI, 0.933-0.950), and in the validation set, the AUC was 0.899 (95% CI, 0.882-0.916) (Figure 3). In stratified models among patients with cirrhosis, the AUC of the validation set was 0.824 (95% CI, 0.740-0.908), and the AUC was 0.893 (95% CI, 0.875-0.911) among patients without cirrhosis. In models stratified by race and ethnicity, AUCs in validation sets were 0.924 (95% CI, 0.903-0.944) in Asian patients, 0.836 (95% CI, 0.734-0.938) in Black patients, 0.942 (95% CI, 0.909-0.975) in Hispanic patients, 0.919 (95% CI, 0.901-0.937) in White patients, and 0.887 (95% CI, 0.833-0.940) in patients who identified as other race or ethnicity. In age-stratified analyses the model performed better in younger populations, with the best performance among patients aged 40 years or younger (AUC, 0.958 [95% CI, 0.931-0.986]), and lowest performance among patients aged 76 years or older (AUC, 0.798 [95% CI, 0.740-0.855]) (Table 2).

Performance metrics were calculated for predicting HCC risk using the 3 categories of low-, medium-, and high-risk. At the medium-risk threshold, which identified 18.6% of the overall sample, the model had a sensitivity of 87.5%, specificity of 81.4%, PPV of 0.2%, NNS of 406, and NPV close to 100%. At the high-risk threshold, which identified 9.9% of the overall sample, the model had a sensitivity of 78.4%, specificity of 90.1%, PPV of 0.4%, NNS of 241, and NPV close to 100%. Based on the algorithm, and balancing sensitivity, specificity, and NNS, those in the high-risk group would be considered for higher intensity surveillance protocols.

In our model, the Hosmer-Lemeshow goodness-of-fit test had a *P* = .99 for the validation set, indicating that our validation model had acceptable calibration and prediction performance. Furthermore, our model had a mean predicted risk of 0.05%, identical to the observed risk of 0.05% in the overall sample, indicating excellent mean calibration (ie, calibration in the large). Our model had a calibration slope of 1.01, indicating that estimated risks were slightly moderate, ie, slightly low for patients who were at high risk and slightly high for patients who were at low risk.^35^ Lastly, graphically, our calibration plot shows the mean predicted vs observed HCC risk in the validation set (543 577 patients). We found that our model fluctuated across the risk range from 0% to 6%, but the estimates were close to the line of equality (Figure 1).

## Discussion

In this large prognostic study, EHR data from 1 811 461 patients in an integrated health care delivery system in Northern California were used to develop a prediction model to discriminate between individuals with and without incident HCC with good precision. The resulting model achieved an excellent AUC by conventional guidelines for the validation set.^36^ This model could be applied to risk stratify adult patients with MASLD who lack other HCC risk factors, including primary biliary cirrhosis, HCV infection, or alcohol-associated liver disease, into low-, medium-, and high-risk groups. The inclusion of predictors routinely available in the EHR allows for this model to be used in primary care settings to identify patients with MASLD at higher risk for HCC who may need close monitoring or surveillance.

Due to low HCC incidence, the algorithm for the high-risk group had a low PPV (0.4) and a high NNS (241). This is a common problem in models predicting rare outcomes.^37^ Therefore, this algorithm is well-suited as a starting point to identify patients with high-risk MASLD who may need HCC surveillance in the near future. One use of this model would be to identify patients with high-risk MASLD for enrollment in trials for prevention and/or surveillance of HCC. Another use would be for clinicians to consider HCC screening only in patients predicted to be in the high-risk group. The algorithm would identify approximately 10% of the MASLD population, greatly reducing the proportion of patients needing screening with ultrasonography, while still capturing more than 78% of those likely to develop HCC in the next 5 to 10 years.

This study adds to the limited body of HCC prediction models among patients with MASLD. Some studies in populations with noncirrhotic MASLD have identified independent genetic risk factors (*PNPLA3*, *TM6SF2*, and *MBOAT7*)^38^ and rare pathogenic variants (*PNPLA3*, *TM6SF2*, *GCKR*, and *MBOAT7*).^10,39,40^ However, only 1 of these studies developed an HCC prediction model, with relatively low predictive performance (AUC, 0.64; sensitivity, 0.43; specificity, 0.80).^10^ Furthermore, genetic markers and pathogenic variants are rarely measured during routine clinical visits, making these mostly unusable outside of research settings. Other noninvasive risk scores in adults with MASLD have been developed using clinical variables. One large Korean study stratified 10-year risk of HCC with an AUC of 0.92,^11^ using age, sex, smoking, diabetes, total cholesterol level, and ALT. In a small European study, Best et al^12^ identified sex, age, and serum levels of AFP, AFP isoform L3, and des-γ-carboxy prothrombin. Best et al^12^ identified prevalent HCC with excellent discrimination (AUC, 0.96); however, they did not test the model’s ability to stratify future risk. In another large study of 18 million patients with MASLD from 4 European cohorts, Alexander et al^25^ found MASH, high-risk FIB-4 scores, and diabetes to be HCC predictors. Additionally, in another small European study, Younes et al^13^ used noninvasive scoring systems (MASLD fibrosis score; FIB-4, BMI, AST/ALT ratio, and diabetes; and aspartate transaminase-platelet ratio index) and the Hepamet fibrosis score to risk stratify patients with MASLD for HCC risk and other liver outcomes. Younes et al^13^ found that MASLD fibrosis score had an excellent discrimination ability (AUC, 0.9) for HCC. Although some of these models had adequate performance, these were developed in homogeneous populations of Korean^11^ or European descent,^10,12,13,25,38,39,40^ which may not generalize to diverse racial and ethnic groups in the US.

To our knowledge, this is the first HCC prediction study developed among racially and ethnically diverse patients in the US. In our study, we found Asian and Hispanic patients had a higher risk of HCC compared with Black and White patients. These findings are largely consistent with prior findings.^41,42^ In analyses stratified by race and ethnicity, our models performed well for each group.

In stratified analyses among patients with cirrhosis, the model had excellent discrimination (AUC, 0.824)^36^ and was superior to models from prior studies, which had AUCs that ranged from 0.64 to 0.76.^43,44,45,46,47^ Similar to our model, 1 or more of these prior studies identified age, sex, race, diabetes, BMI, platelet count, AST, ALT, bilirubin, and albumin as independent HCC predictors. Current guidelines recommend HCC surveillance in patients with cirrhosis, because it improves overall survival.^48^ Therefore, our model could be used to risk stratify patients with MASH cirrhosis to identify patients at greater risk for HCC, even before they develop overt disease manifestations, for prevention efforts or early detection, with a similar or better performance than prior models.

### Limitations

This study has some limitations. First, data came from 1 integrated health care system; although the population was broadly representative of the overall US population,^14,49^ our findings may not adequately generalize to the US population, especially uninsured adults. Second, our model needs to be externally validated before being implemented. Third, this study may have missed some patients with MASLD who developed HCC, especially those with infrequent exposure to the health care system, for whom data was missing. Fourth, due to missing imaging for many participants, there was some misclassification of MASLD ascertainment by using the DSI. In particular, the study incorrectly included some patients without MASLD (false positives), possibly resulting in a lower HCC incidence rate than expected compared with a prior study among patients with MASLD in the Department of Veterans Affairs,^42^ and this may have influenced the model’s performance. Fifth, our model may not perform as well in populations with a lower prevalence of HBV infection. Sixth, our model did not account for death as a competing event, which can overestimate the cumulative incidence^50^; however, given that our sample had a relatively low mortality rate (<1% per year), we would expect the overestimation of the cumulative incidence to be minimal. Seventh, we lacked gut permeability or microbiome composition, which have been identified as risk factors for HCC in patients with MASLD^8^; however, these data are not routinely available, and including them as predictors would limit the usability of this model in clinical practice. Strengths of this study include the large racially and ethnically diverse sample, the use of individual-level EHR data, and the long longitudinal follow-up.

## Conclusions

To our knowledge, this prognostic study presents the first HCC risk algorithm for racially and ethnically diverse patients with MASLD in the US using routinely collected EHR variables that adequately discriminated among patients at low-, medium-, and high-risk for developing HCC. This model can serve as a starting point to identify patients with MASLD and guide decision-making about risk stratifying patients at high risk of HCC, whether for prevention efforts or higher-intensity HCC surveillance.

## Notes

Supplement 1. **eTable 1.** Identification of Prevalent Metabolic Dysfunction-Associated Steatotic Liver Disease **eTable 2.** *ICD-9* and *ICD-10* Diagnosis Codes Used to Identify HCC and Comorbidities **eFigure.** Cumulative Incidence Curves Showing the Cumulative Incidence of HCC in a Cohort of Patients With MASLD Followed Up From 2009 to September 30, 2021

Supplement 2.
Data Sharing Statement
