# Supplementary material for: Hepatocellular Carcinoma in Metabolic Dysfunction-Associated Steatotic Liver Disease
Source: JAMA Netw Open. 2024 Jul 11;7(7):e2421019. doi: 10.1001/jamanetworkopen.2024.21019 (PMC11240192; doi:10.1001/jamanetworkopen.2024.21019)
Supplement: Supplement 1. — eTable 1. Identification of Prevalent Metabolic Dysfunction-Associated Steatotic Liver Disease eTable 2. ICD-9 and ICD-10 Diagnosis Codes Used to Identify HCC and Comorbidities eFigure. Cumulative Incidence Curves Showing the Cumulative Incidence of HCC in a Cohort of Patients With MASLD Followed Up From 2009 to September 30, 2021 [file jamanetwopen-e2421019-s001.pdf]

Supplemental Online Content

Rodriguez LA, Schmittdiel JA, Liu L, et al. Hepatocellular carcinoma in metabolic dysfunction-associated steatotic liver disease. *JAMA Netw Open*. 2024;7(7):e2421019. doi:10.1001/jamanetworkopen.2024.21019

eTable 1. Identification of Prevalent Metabolic Dysfunction-Associated Steatotic Liver Disease

eTable 2. *ICD-9* and *ICD-10* Diagnosis Codes Used to Identify HCC and Comorbidities

eFigure. Cumulative Incidence Curves Showing the Cumulative Incidence of HCC in a Cohort of Patients With MASLD Followed Up From 2009 to September 30, 2021

This supplemental material has been provided by the authors to give readers additional information about their work.

© 2024 Rodriguez LA et al. *JAMA Network Open.*

eTable 1. Identification of Prevalent Metabolic Dysfunction-Associated Steatotic Liver Disease

Data Sources	MASLD*
**ICD-based**<br>• Outpatient encounter<br>• Hospital discharge<br>• Problem list	ICD-9-CM diagnosis codes: 571.9 (chronic liver disease NOS)
	ICD-10-CM diagnosis codes: K76.0 (fatty [change of] liver, NEC), K73.8 (other hepatitis, NEC), K75.81 (Nonalcoholic steatohepatitis)
**Imaging-based**<br>Imaging studies of interest based on CPT-4 codes:<br>• Ultrasound: 76700, 76705<br>• Computerized tomography: 74150, 74160, 74170, 74175, 74176, 74177, 74178<br>• Magnetic resonance imaging: 74181, 74182, 74183	Using SAS regular expression to search for terms of interest in “Impression Section” in members with completed imaging studies:<br>• Fatty liver<br>• Hepatic steatosis<br>• Increased liver echogenicity<br>Terms negating MASLD of interest:<br>• Hepatitis C<br>• Autoimmune hepatitis<br>• Primary biliary cirrhosis
**Dallas Steatosis Index-based**<br>• Completed laboratory tests: Triglyceride, Alanine aminotransferase (ALT)<br>• Body mass index	Dallas Steatosis Index (DSI) calculated as:<br>−9.4 + 0.316 (if ≥50 years-of-age and female) + 2.4 (if known diabetes) + 0.02 (equals 0 if diabetic; if not diabetic equals glucose concentration in mg/dL) + 0.3 (if known hypertension) + 0.5 (if Hispanic/Asian/Other Race/Ethnicity) + Ln (triglycerides in mg/dL) + 0.4 (if ALT 13.5−19.49 IU/L) + 1.1 (if ALT 19.5−40 IU/L) + 1.5 (if ALT >40 IU/L) + 0.7 (if not black and BMI 25−27.49 kg/m^2) + 1.4 (if not black and BMI 27.5−34.9 kg/m^2) + 1.9 (if not black and BMI 35−37.49 kg/m^2) + 2.6 (if not black and BMI >37.5 kg/m^2) - 0.2 (if black and BMI 25−27.49 kg/m^2) + 0.9 (if black and BMI 27.5−34.9 kg/m^2) + 0.8 (if black and BMI 35−37.49 kg/m^2) + 1.8 (if black and BMI >37.5 kg/m^2)<br>Source: https://doi.org/10.1016/j.cgh.2020.01.020

Abbreviations: ALT, alanine transaminase; BMI, body mass index; CPT, current procedural terminology; ICD, International Classification of Diseases; MASLD, metabolic dysfunction-associated steatotic liver disease; NEC, not elsewhere classified; NOS, not otherwise specified.

* Excluding hepatitis C (ICD-9-CM: 070.41, 070.44, 070.51, 070.54, 070.70, 070.71, V02.62; ICD-10-CM: B17.10, B18.2, Z86.19); autoimmune hepatitis (ICD-9-CM: 571.42 ICD-10-CM: K75.4); primary biliary cirrhosis (ICD-9-CM: 571.6 ICD-10-CM: K74.5); acute alcoholic hepatitis (ICD-9: 571.1; ICD-10: K70.10, K70.11); alcoholic fatty liver (ICD-9: 571.0; ICD-10: K70.0); unspecified alcoholic liver damage (ICD-9: 571.3; ICD-10: K70.40, K70.41, K70.9); alcoholic cirrhosis (ICD-9: 571.2; ICD-10: K70.2, K70.30, K70.31); alcohol use disorder (ICD-9: 305.00–305.03; ICD-10: F10.1x); and chemical dependency treatment (ICD-9: 304.61-6, 304.70-72, 304.80-82, 304.90-92; ICD-10: F19.20).

© 2024 Rodriguez LA et al. *JAMA Network Open.*

eTable 2. *ICD-9* and *ICD-10* Diagnosis Codes Used to Identify HCC and Comorbidities

Condition	ICD-9/10 code(s) used and definition(s)
Cirrhosis	ICD9/10 codes of 456.1, 456.21, 571.2, 571.5, I85.00, I98.2, K70.3, K71.7, K74.6, 572.2, 572.3, 572.4, 456.0, 456.20, 782.4, 789.5, I85.01, I86.4, I98.20, I98.3, K72.1, K72.9, K76.6, K76.7, R17, R18, or FIB4 score ≥3.25
Drug use	292, 292.0, 292.1, 292.11, 292.12, 292.2, 292.8, 292.81, 292.82, 292.83, 292.84, 292.85, 292.89, 292.9, 304, 304.0, 304.00, 304.01, 304.02, 304.03, 304.1, 304.10, 304.11, 304.12, 304.13, 304.2, 304.20, 304.21, 304.22, 304.23, 304.3, 304.30, 304.31, 304.32, 304.33, 304.4, 304.40, 304.41, 304.42, 304.43, 304.5, 304.50, 304.51, 304.52, 304.53, 304.6, 304.60, 304.61, 304.62, 304.63, 304.7, 304.70, 304.71, 304.72, 304.73, 304.8, 304.80, 304.81, 304.82, 304.83, 304.9, 304.90, 304.91, 304.92, 304.93, 305.2, 305.20, 305.21, 305.22, 305.23, 305.3, 305.30, 305.31, 305.32, 305.33, 305.4, 305.40, 305.41, 305.42, 305.43, 305.5, 305.50, 305.51, 305.52, 305.53, 305.6, 305.60, 305.61, 305.62, 305.63, 305.7, 305.70, 305.71, 305.72, 305.73, 305.8, 305.80, 305.81, 305.82, 305.83, 305.9, 305.90, 305.91, 305.92, 305.93, F11, F11.1, F11.10, F11.11, F11.12, F11.120, F11.121, F11.122, F11.129, F11.14, F11.15, F11.150, F11.151, F11.159, F11.18, F11.181, F11.182, F11.188, F11.19, F11.2, F11.20, F11.21, F11.22, F11.220, F11.221, F11.222, F11.229, F11.23, F11.24, F11.25, F11.250, F11.251, F11.259, F11.28, F11.281, F11.282, F11.288, F11.29, F11.9, F11.90, F11.92, F11.920, F11.921, F11.922, F11.929, F11.93, F11.94, F11.95, F11.950, F11.951, F11.959, F11.98, F11.981, F11.982, F11.988, F11.99, F12, F12.1, F12.10, F12.11, F12.12, F12.120, F12.121, F12.122, F12.129, F12.15, F12.150, F12.151, F12.159, F12.18, F12.180, F12.188, F12.19, F12.2, F12.20, F12.21, F12.220, F12.221, F12.222, F12.229, F12.23, F12.25, F12.250, F12.251, F12.259, F12.28, F12.280, F12.288, F12.29, F12.9, F12.90, F12.92, F12.920, F12.921, F12.922, F12.929, F12.93, F12.95, F12.950, F12.951, F12.959, F12.98, F12.980, F12.988, F12.99, F13, F13.1, F13.10, F13.11, F13.12, F13.120, F13.121, F13.129, F13.14, F13.15, F13.150, F13.151, F13.159, F13.18, F13.180, F13.181, F13.182, F13.188, F13.19, F13.2, F13.20, F13.21, F13.22, F13.220, F13.221, F13.229, F13.23, F13.230, F13.231, F13.232, F13.239, F13.24, F13.25, F13.250, F13.251, F13.259, F13.26, F13.27, F13.28, F13.280, F13.281, F13.282, F13.288, F13.29, F13.9, F13.90, F13.92, F13.920, F13.921, F13.929, F13.93, F13.930, F13.931, F13.932, F13.939, F13.94, F13.95, F13.950, F13.951, F13.959, F13.96, F13.97, F13.98, F13.980, F13.981, F13.982, F13.988, F13.99, F14, F14.1, F14.10, F14.11, F14.12, F14.120, F14.121, F14.122, F14.129, F14.14, F14.15, F14.150, F14.151, F14.159, F14.18, F14.180, F14.181, F14.182, F14.188, F14.19, F14.2, F14.20, F14.21, F14.22, F14.220, F14.221, F14.222, F14.229, F14.23, F14.24, F14.25, F14.250, F14.251, F14.259, F14.28, F14.280, F14.281, F14.282, F14.288, F14.29, F14.9, F14.90, F14.92, F14.920, F14.921, F14.922, F14.929, F14.94, F14.95, F14.950, F14.951, F14.959, F14.98, F14.980, F14.981, F14.982, F14.988, F14.99, F15, F15.1, F15.10, F15.11, F15.12,

© 2024 Rodriguez LA et al. *JAMA Network Open.*

	F15.120, F15.121, F15.122, F15.129, F15.14, F15.15, F15.150, F15.151, F15.159, F15.18, F15.180, F15.181, F15.182, F15.188, F15.19, F15.2, F15.20, F15.21, F15.22, F15.220, F15.221, F15.222, F15.229, F15.23, F15.24, F15.25, F15.250, F15.251, F15.259, F15.28, F15.280, F15.281, F15.282, F15.288, F15.29, F15.9, F15.90, F15.92, F15.920, F15.921, F15.922, F15.929, F15.93, F15.94, F15.95, F15.950, F15.951, F15.959, F15.98, F15.980, F15.981, F15.982, F15.988, F15.99, F16, F16.1, F16.10, F16.11, F16.12, F16.120, F16.121, F16.122, F16.129, F16.14, F16.15, F16.150, F16.151, F16.159, F16.18, F16.180, F16.183, F16.188, F16.19, F16.2, F16.20, F16.21, F16.22, F16.220, F16.221, F16.229, F16.24, F16.25, F16.250, F16.251, F16.259, F16.28, F16.280, F16.283, F16.288, F16.29, F16.9, F16.90, F16.92, F16.920, F16.921, F16.929, F16.94, F16.95, F16.950, F16.951, F16.959, F16.98, F16.980, F16.983, F16.988, F16.99, F18, F18.1, F18.10, F18.11, F18.12, F18.120, F18.129, F18.14, F18.15, F18.150, F18.151, F18.159, F18.17, F18.18, F18.180, F18.188, F18.19, F18.2, F18.20, F18.21, F18.22, F18.220, F18.221, F18.229, F18.24, F18.25, F18.250, F18.251, F18.259, F18.27, F18.28, F18.280, F18.288, F18.29, F18.9, F18.90, F18.92, F18.920, F18.921, F18.929, F18.94, F18.95, F18.950, F18.951, F18.959, F18.97, F18.98, F18.980, F18.988, F18.99, F19, F19.1, F19.10, F19.11, F19.12, F19.120, F19.121, F19.122, F19.129, F19.14, F19.15, F19.150, F19.151, F19.159, F19.16, F19.17, F19.18, F19.180, F19.181, F19.182, F19.188, F19.19, F19.2, F19.20, F19.21, F19.22, F19.220, F19.221, F19.222, F19.229, F19.23, F19.230, F19.231, F19.232, F19.239, F19.24, F19.25, F19.250, F19.251, F19.259, F19.26, F19.27, F19.28, F19.280, F19.281, F19.282, F19.288, F19.29, F19.9, F19.90, F19.92, F19.920, F19.921, F19.922, F19.929, F19.93, F19.930, F19.931, F19.932, F19.939, F19.94, F19.95, F19.950, F19.951, F19.959, F19.96, F19.97, F19.98, F19.980, F19.981, F19.982, F19.988, F19.99
Hepatocellular carcinoma (HCC)	KPNC Tumor Registry
Human immunodeficiency virus (HIV)	ICD-9 042 to 044.999 inclusive, or ICD-10 B20 to B20.999 inclusive
Metabolic dysfunction-associated steatotic liver disease (MASLD)	ICD-9-CM diagnosis codes: 571.9 (chronic liver disease NOS)<br>ICD-10-CM diagnosis codes: K76.0 (fatty [change of] liver, NEC), K73.8 (other hepatitis, NEC), K75.81 (Nonalcoholic steatohepatitis)
Other immune disease	275.1, E83.01, 275.01, 275.03, E83.110, 273.4, E88.01, 571.5, K74.69
Steatohepatitis	571.1, 571.2, 571.8, K70.10, K70.11, K70.2, K70.30, K70.31, K75.81

Abbreviations: HCC, hepatocellular carcinoma; HIV, human immunodeficiency virus; ICD, International Classification of Diseases; INR, international normalized ratio; KPNC, Kaiser Permanente Northern California; MASLD, metabolic dysfunction-associated steatotic liver disease; NEC, not elsewhere classified; NOS, not otherwise specified.

© 2024 Rodriguez LA et al. *JAMA Network Open.*

eFigure. Cumulative Incidence Curves Showing the Cumulative Incidence of HCC in a Cohort of Patients With MASLD Followed-Up From 2009 to September 30, 2021
Plotted by low-, medium- and high-risk intervals of HCC risk. HCC, hepatocellular carcinoma; MASLD, metabolic dysfunction-associated steatotic liver disease.

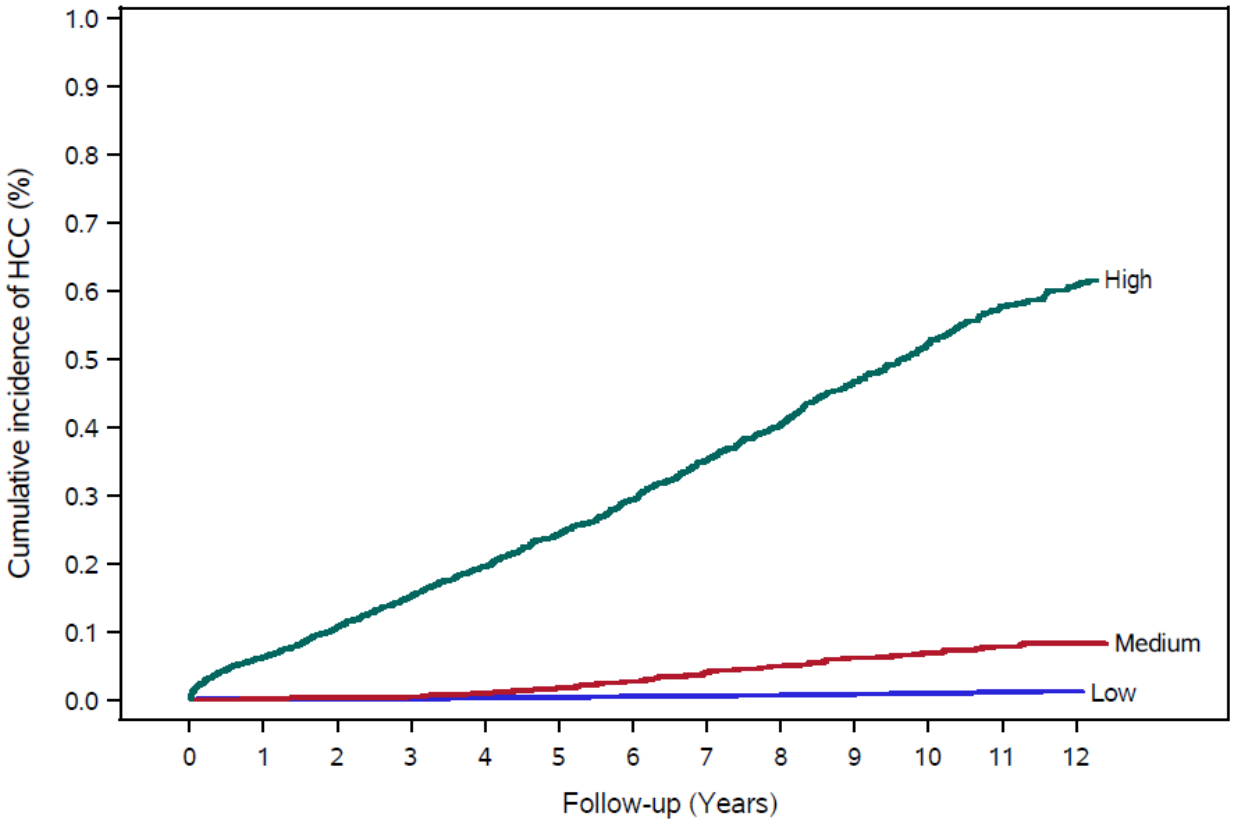

N° at risk						
High	178,930	159,250	131,660	103,778	77,539	48,909
Medium	157,472	146,609	124,772	99,716	75,013	45,808
Low	1,475,059	1,427,067	1,217,530	933,040	659,332	330,536

© 2024 Rodriguez LA et al. *JAMA Network Open.*
