# Supplementary material for: Hepatocellular Carcinoma in Metabolic Dysfunction-Associated Steatotic Liver Disease
Source: JAMA Netw Open. 2024 Jul 11;7(7):e2421019. doi: 10.1001/jamanetworkopen.2024.21019 (PMC11240192; doi:10.1001/jamanetworkopen.2024.21019)
Supplement: Supplement 2. — Data Sharing Statement [file jamanetwopen-e2421019-s002.pdf]

Data Sharing Statement

Rodriguez. Hepatocellular Carcinoma in Metabolic Dysfunction-Associated Steatotic Liver Disease. *JAMA Netw Open*. Published July 11, 2024.
doi:10.1001/jamanetworkopen.2024.21019

Data
Data available: No
